# Myeloperoxidase-Dependent LDL Modifications in Bloodstream Are Mainly Predicted by Angiotensin II, Adiponectin, and Myeloperoxidase Activity: A Cross-Sectional Study in Men

**DOI:** 10.1155/2013/750742

**Published:** 2013-11-18

**Authors:** Karim Zouaoui Boudjeltia, Cédric Delporte, Pierre Van Antwerpen, Thierry Franck, Didier Serteyn, Nicole Moguilevsky, Martine Raes, Luc Vanhamme, Michel Vanhaeverbeek, Alain Van Meerhaeghe, Thierry Roumeguère

**Affiliations:** ^1^Laboratory of Experimental Medicine (ULB 222 Unit), CHU de Charleroi, A. Vésale Hospital, Université Libre de Bruxelles, Rue de Gozée 706, 6110 Montigny-le-Tilleul, Belgium; ^2^Laboratory of Pharmaceutical Chemistry and Analytical Platform, Faculty of Pharmacy, Université Libre de Bruxelles, Boulevard du Triomphe, Campus Plaine CP 205/5, 1050 Brussels, Belgium; ^3^Center for Oxygen Research and Development, Institute of Chemistry B6a, University of Liège, 4000 Liège, Belgium; ^4^Technology Transfer Office, University of Namur, rue de Bruxelles 61, 5000 Namur, Belgium; ^5^Laboratory of Biochemistry and Cellular Biology (NARILIS), University of Namur, rue de Bruxelles, 61, 5000 Namur, Belgium; ^6^Institute for Molecular Biology and Medicine (IBMM), Université Libre de Bruxelles, Rue des Professeurs Jeener et Brachet 12, 6041 Gosselies, Belgium; ^7^Department of Urology, Erasme University Hospital, Université Libre de Bruxelles, Route de Lennik 808, 1070 Brussels, Belgium

## Abstract

The present paradigm of atherogenesis proposes that low density lipoproteins (LDLs) are trapped in subendothelial space of the vascular wall where they are oxidized. Previously, we showed that oxidation is not restricted to the subendothelial location. Myeloperoxidase (MPO), an enzyme secreted by neutrophils and macrophages, can modify LDL (Mox-LDL) at the surface of endothelial cells. In addition we observed that the activation of the endothelial cells by angiotensin II amplifies this process. We suggested that induction of the NADPH oxidase complex was a major step in the oxidative process. Based on these data, we asked whether there was an independent association, in 121 patients, between NADPH oxidase modulators, such as angiotensin II, adiponectin, and levels of circulating Mox-LDL. Our observations suggest that the combination of blood angiotensin II, MPO activity, and adiponectin explains, at least partially, serum Mox-LDL levels.

## 1. Introduction

Atherosclerosis is an inflammatory disease involving a crosstalk between vascular cells, monocytes, proinflammatory cytokines, chemokines, and growth factors [1–3]. The current paradigm of early atherosclerosis claims that low-density lipoprotein (LDL) particles are trapped in the subendothelial space of the vascular wall where they can be oxidized. The precise physiological process for LDL oxidation *in vivo* is still largely unknown and the occurrence of LDL oxidation outside the lesion sites has not definitively been ruled out yet.

Evidence accumulated during the last decade has suggested implication of myeloperoxidase (MPO) in inflammation leading to atherogenesis. MPO is produced by macrophages and neutrophils [4] and via its chlorination activity, MPO produces hypochlorous acid (HOCl) from hydrogen peroxide (H_2_O_2_) and chloride anion (Cl^−^). HOCl can oxidize protein-bound amino acid residues among which the formation of 3-chlorotyrosine is considered as specific of the activity of MPO as the latter is the only human enzyme able to produce HOCl. In the context of atherogenesis, MPO, 3-chlorotyrosine, and MPO-dependent modified LDL (Mox-LDL) have all been detected in human atherosclerotic lesions and in the bloodstream [5–8]. 

We previously demonstrated that Mox-LDL generation could occur *in vitro* at the surface of the endothelial cells suggesting that it was not restricted to the subendothelial space *in vivo* [9]. The triad made up by endothelial cell, circulating LDL and MPO, allowed a synergic mechanism for producing Mox-LDL. The starting point of this reaction is the generation of superoxide anion (O_2_^−^) by the membrane bound nicotinamide-adenine-dinucleotide phosphate (NADPH) oxidase. O_2_^−^ is further dismutated into H_2_O_2_ a substrate for MPO to produce HOCl. We recently reported, in two different clinical situations, that this indeed enables MPO to rapidly modify LDL and serum proteins by oxidations [8, 10]. Furthermore, NADPH oxidase is activated and upregulated by angiotensin II (ANGII) via the ANGII type I (AT1) receptor present at the surface of endothelial cells [11]. This enzymatic complex therefore plays a central role in the Mox-LDL generation [9]. 

Based on these data, we wondered whether there was an independent association between NADPH oxidase modulators, such as ANGII, adiponectin [12], and levels of circulating Mox-LDL. To test this hypothesis, we report the data observed in a cohort of male patients (*n* = 121) consulting for lower urinary tract symptoms (LUTS). Indeed, LUTS is associated with the erectile dysfunction, which is an early predictive sign for atherosclerotic cardiovascular events [13]. 

## 2. Material and Methods

### 2.1. Patients

Subjects were 121 males with a mean age of 58.8 ± 10.8 who consulted for lower urinary tract symptoms (LUTS) at the Erasme University Hospital. This study conforms with the Declaration of Helsinki and its protocol was approved by the Ethics Committee of the Erasme University Hospital. Finally, all subjects gave their written informed consent.

### 2.2. Standard Analyses

Blood samples were centrifuged for 10 minutes at 4000 g and the supernatant was collected and frozen. Blood tests were performed at the Laboratory of Experimental Medicine of the University Hospital of Charleroi, Site A. Vésale, Unit 222, ULB. The following parameters were measured: C-reactive protein (CRP), blood glucose, total cholesterol, triglycerides, HDL-cholesterol (standard laboratory techniques PLC), and adiponectin. LDL-cholesterol levels were calculated using the Friedewald formula (LDL-chol (mg/dL) = T-chol-HDL-chol-TG/5).

### 2.3. MPO and Mox-LDL Analyses

Mox-LDL was measured using a sandwich ELISA kit [9]. The specificity of the antibody was further assessed by analyzing LDL oxidized with peroxynitrite (0, 10, 100, and 1000 *μ*M) and comparing with LDL oxidized by MPO/hydrogen peroxide/chloride. Other oxidants produced by MPO such as HOSCN/^−^OSCN, HOBr/^−^OBr, and HOI/^−^OI (from MPO/hydrogen peroxide/corresponding halide) were also used to oxidize LDL and to test the specificity of LDL. It resulted in the fact that the antibody is highly specific for Mox-LDL.

The active and total MPO contents in plasma were measured using the licensed SIEFED and ELISA (ELIZEN MPO, Zentech SA, Belgium) methods [14]. By using these two techniques, we are able to distinguish active and total MPO contents in plasma and to determine the specific activity of MPO (MPO activity/MPO antigen ratio). 

### 2.4. Angiotensin II, Adiponectin, and Interleukin-8 Analyses

ANGII was determined in plasma by a radioimmunoassay kit (BioSource, Nivelles, Belgium). IL-8 and adiponectin concentrations were quantified using ELISA tests (Becton Dickinson). 

### 2.5. Statistic

Data were analyzed using the SigmaPlot 12.0 software (Systat, San Jose, CA). Results were considered statistically significant with a two-tailed *P* < 0.05. Two models of multiple linear regression analysis were tested using a backward stepwise selection of explicative variables. 

## 3. Results and Discussion

The purpose of the present study was to explore whether there is an independent association between ANGII (an NADPH modulator), adiponectin, and levels of circulating Mox-LDL. In this context we analyzed various parameters within 121 male subjects who consulted for the first time for LUTS.  Table 1 shows the means and SD of the parameters measured or calculated within patients.

Table 2 describes two models of multivariate analysis of backward regression in these subjects. The standardized regression coefficients are given for each model. As shown in Table 2, in the first model (Model 1) we set Mox-LDL as the dependent variable, while the independent variables included the parameters described above. Significant linear correlations were found between Mox-LDL levels and ANGII, and MPO activity (both positively) and also adiponectin content (negatively). In the second model (Model 2) the Mox-LDL/ApoB ratio (an estimation of the proportion of MPO-modified LDL in the bloodstream) was set as dependent variable and the same set of above parameters as independent variables. The same variables as in Model 1 were found to predict the Mox-LDL/ApoB ratio.

Our observations suggest that the combination of blood ANGII, MPO, activity and adiponectin explains at least partially the serum Mox-LDL levels. They corroborate and extend our previous data showing that oxidation could also take place at the surface of endothelial cells [9, 15] and that plasma level of Mox-LDL follows the level of MPO in patients during a hemodialysis process [15, 16]. They are underpinned by established physiopathological mechanisms as endothelial cells express NADPH oxidase, the activity and expression of which are increased by ANGII binding to the AT1 receptor [12]. In support of our proposal, we previously observed that hypertensive COPD patients treated by angiotensin-converting enzyme inhibitors had reduced levels of circulating Mox-LDL (our unpublished data). This is an alternative and complementary explanation to the common model positing that the presence of modified LDL in the circulation is due to the back diffusion of modified LDL from the vessel to the circulation and is a marker of plaque instability in patients with coronary artery disease. Furthermore, it recently arose that human peroxidasin 1, also called vascular peroxidase 1 (VPO1), might be involved in the *in vivo* production of HOCl and so potentially contributes to the oxidation of LDL [17]. Moreover, VPO1 was also suggested as an inductor of vascular smooth muscle cell proliferation [18]. However, further experiments are needed as the formation of HOCl by VPO1 is low at physiological pH. 

We also uncovered a negative linear correlation between oxidative stress and adiponectin in our multiple linear regression models (Table 2). This is in agreement with the observation that adiponectin reduced *in vitro* and *in vivo* the NADPH oxidase activity and hence oxidative stress [12]. It is also in support of the general agreement that adiponectin, which is secreted by fat tissue, is antiatherogenic by modulating cytokine inflammatory cascades and inhibiting cholesterol incorporation.

In sum, our study suggests that the combined action of ANGII, MPO, and adiponectin might explain the serum Mox-LDL levels. A definitive validation or invalidation of the proposed role of ANGII in the generation of serum Mox-LDL will request a double blind randomized crossover study comparing subjects receiving an angiotensin-converting enzyme inhibitor or an angiotensin II receptor antagonist and a placebo.

## Figures and Tables

**Table 1:** Patient parameters.

*N* = 121	Mean	SD
Age (years)	59	11
BMI (kg/m^2^)	26	4
Glycemia (mg/dL)	107	48
Total cholesterol (mg/dL)	209	39
Triglycerides (mg/dL)	184	142
HDL cholesterol (mg/dL)	53	18
LDL cholesterol (mg/dL)	122	35
Interleukin-8 (ng/mL)	17	10
Mox-LDL (*µ*g/mL)	9	13
Mox-LDL/ApoB (*µ*g/mg)	0.1	0.2
Angiotensin II (pmol/L)	14	11
Adiponectin (ng/mL)	5252	4197
MPO activity (MPOA) (mU/mL)	27	18
MPO antigen (MPOAg) (ng/mL)	50	36
MPOA/MPOAg (mU/ng)	0.6	0.2

Conversion for lipids: total cholesterol, HDL-c and LDL-c: 1 mmol/L = 38.67 mg/dL.

Triglycerides: 1 mmol/L = 38.67 mg/dL.

BMI: body mass index.

**Table 2:** Multivariate analysis of backward regressions in the total population.

*N* = 121	Standardized regression coefficient	*P* value
Model 1 *R* = 0.52, *F* = 9.7, P < 0.001		
ANGII	0.425	<0.001
MPO activity	0.236	0.018
Adiponectin	−0.196	0.05
Model 2 *R* = 0.58, *F* = 12.6, *P* < 0.001		
ANGII	0.446	<0.001
MPO activity	0.315	0.001
Adiponectin	−0.201	0.03

Parameters introduced in the stepwise multiple regression analysis: age, BMI, adiponectin, IL-8, total cholesterol, HDL-c, LDL-c, triglycerides, glycaemia, MPO antigen, MPO activity, and the ratio MPO activity/MPO antigen. Model 1: the Mox-LDL is the dependent variable. Model 2: the Mox-LDL/ApoB is the dependent variable.

## Abbreviations

ANGII: Angiotensin II
ApoB-100: Apolipoprotein B-100
LDL: Low-density lipoprotein
LUTS: Lower urinary tract symptoms
MPO: Myeloperoxidase
Mox-LDL: Low-density lipoprotein modified by the MPO-H_2_O_2_-chloride system
NADPH: Nicotinamide-adenine-dinucleotide phosphate.
